# Rheological and Tribological Properties of Nanocellulose-Based Ecolubricants

**DOI:** 10.3390/nano11112987

**Published:** 2021-11-06

**Authors:** Samuel D. Fernández-Silva, Miguel A. Delgado, Claudia Roman, Moisés García-Morales

**Affiliations:** Departamento de Ingeniería Química, Centro de Investigación en Tecnología de Productos y Procesos Químicos (Pro2TecS), Campus de “El Carmen”, Universidad de Huelva, 21071 Huelva, Spain; samuel.fernandez@diq.uhu.es (S.D.F.-S.); claudia.roman@diq.uhu.es (C.R.); moises.garcia@diq.uhu.es (M.G.-M.)

**Keywords:** sustainability, lubrication, friction coefficient, wear, nanocellulose, vegetable oil

## Abstract

Based on the response surface methodology, a rheological and tribological study carried out on eco-friendly lubricants is described. Such ecolubricants consisted of fibrillated or crystalline nanocellulose in vegetable oil (castor oil, high oleic sunflower oil or their mixtures). Cellulose nanoparticles showed noticeable friction-reducing and anti-wear properties within the boundary and mixed lubrication regimes, which were found to be dependent on nanocellulose concentration, base oil composition and applied normal force. In general, both types of nanocellulose performed equally well. An excellent tribological performance, with large wear scar diameter reductions, was achieved with 3.3 wt.% (or higher) nanocellulose dispersions in castor oil-rich mixtures. The observed behavior was explained on the basis of enhanced viscosity of castor oil-rich suspensions and the preferential action of the most polar components, nanocellulose and ricinoleic acid, in the vicinity of the contact surfaces.

## 1. Introduction

Lubricants are substances that help to reduce friction and wear between two surfaces in contact with a relative motion. They are essential for industrial and non-industrial machinery, including every application with moving parts [1]. Liquid lubricants are mainly composed of either mineral-based or synthetic oil. Additives are also used to impart specific properties to the oil. On these grounds, the inclusion of nanoparticles in lubricant formulations is of especial interest. Dispersions of nanoparticles in a base oil are referred to as nanolubricants. Nanoparticles’ action mechanisms in lubricity, which very much depend on their morphology, have resulted in great improvements as far as friction-improving and wear-reducing properties are concerned [2,3,4,5,6]. Particularly, these mechanisms include physical protection by forming tribofilms in contact surfaces, as well as rolling, mending and polishing effects [7,8,9]. Various types of nanoparticles used as lubricant additives to improve friction, wear and extreme pressure properties have been reported. Among these, metals, such as Cu, Fe and Co [10,11], and metal oxide nanoparticles, such as CuO, ZnO, TiO_2_, SiO_2_ and Al_2_O_3_ [6,12,13,14,15], have shown anti-friction and anti-wear properties. However, the use of mineral and synthetic oils and hazardous nanoparticles may entail serious damage to both human health and the environment.

Hence, the current concern about the leakage and/or disposal of lubricants to the environment [16] has intensified the search for sustainable formulations, mainly based on vegetable oils and non-toxic additives. Vegetable oils have a lower volatility and are less toxic for the environment than mineral and synthetic oils. Some vegetable oils, such as castor oil (CO) and high oleic sunflower oil (HOSO), may have potential to be used as bases for lubricant formulations. Castor oil is a vegetable oil with unusually high viscosity and other intrinsic properties very suited for lubricant application. It shows good performance at low temperatures, low volatility, high kinematic viscosity and excellent lubrication properties due to H-bonding among the pending hydroxyl groups present in the ricinoleic fatty acid molecules, its main component [17,18]. High oleic sunflower oil, in turn, has low volatility and better thermal resistance than other vegetable oils, thus being a priori another suitable candidate as feedstock for lubricant formulations [19].

Studies bound to improve the tribological performance of vegetable oils by the addition of lignocellulosic materials have led to fully sustainable lubricant formulations. Thus, the performance of cellulose derivative-based oleogels and gel-like dispersions of industrial cellulose pulps was evaluated by Sánchez et al. [20] and Núñez et al. [21], respectively. In addition, Gallego et al. [22] and Borrero-López et al. [23] reported the preparation of oleogels using isocyanate-functionalized lignocellulosic materials for the same use. Moreover, Triviño et al. [24] and Delgado et al. [25] carried out the epoxidation of alkali lignin from cellulose pulp, which they then dispersed in castor oil resulting in gel-like systems with lubricant properties. All these previous studies demonstrated, in general, the potential of lignocellulosic materials, with or without chemical treatment, in the manufacture of lubricating greases with enhanced tribological properties. However, their application in liquid lubricants is constrained by their poor dispersibility, which has been reported to provoke viscous flow instabilities [26].

Nanocellulose may be more appropriate in liquid lubricant formulations. In that sense, an attempt was made to enhance the tribological behavior and thermal properties of an SAE 40 engine oil by adding a hybrid of cellulose nanocrystal (CNC) and copper (II) oxide (CuO) [27]. Frictional force and wear rate were reduced, whilst thermal conductivity and specific heat capacity were increased. Amirruddin [28] pointed out that hybrids of CNC and aluminum oxide (Al_2_O_3_) can produce a reduction in the friction coefficient of up to 16% and in the wear rate of up to 71% when added to a 10 W-40 engine oil. The lubricant was also seen to extend the life of the engine mechanical components. Moreover, Awang et al. [29] showed that the addition of CNC to SAE 40 engine oil significantly reduced the friction and wear rate under all lubrication conditions. Even so, there is still scarce evidence on the use of cellulose nanoparticles in the manufacture of fully sustainable liquid lubricant formulations, where their dispersibility in vegetable oil plays a role in the expected tribological performance [30].

In the present research work, we explored the use of cellulose nanoparticles as a green additive in the formulation of fully biodegradable vegetable-based lubricating oils. Particular emphasis was placed on the role of base oil composition in terms of its contribution to both the friction coefficient and wear scar reductions within the mixed friction regime. Based on that, a set of vegetable oil mixtures loaded with either fibrillated or crystalline nanocelluloses (CNF or CNC, respectively) was prepared and examined, according to the so-called response surface methodology (RSM).

## 2. Materials and Methods

### 2.1. Materials

Crystalline nanocellulose (CNC) and fibrillated nanocellulose (CNF), both supplied by Nanografi Co. Ltd. (Jena, Germany), were used as the particulate phase. CNC, produced by sulfuric acid hydrolysis, has a diameter of 10 to 20 nm and a length of 0.3 to 0.9 µm. In turn, CNF was produced by high-shear mechanical treatment and presents a similar diameter to CNC but larger lengths of 2 to 3 µm. The nanocelluloses were used in their “as-received” form, i.e., dry powder (ca. 4 wt.% moisture).

Castor oil (CO), high oleic sunflower oil (HOSO) and their mixtures were used as dispersing medium. CO, with a kinematic viscosity of 242.5 cSt at 40 °C, was purchased from Guinama (Valencia, Spain). HOSO, with 85 wt.% oleic acid and a kinematic viscosity of 38.5 cSt at 40 °C, was kindly supplied by “Instituto de la Grasa”, CSIC (Seville, Spain). The properties and compositional data for both oils can be found in the literature [17,18]. Vegetable oil mixtures were prepared according to the mixing rule described in [18].

### 2.2. Experimental Design

**Figure 1:**
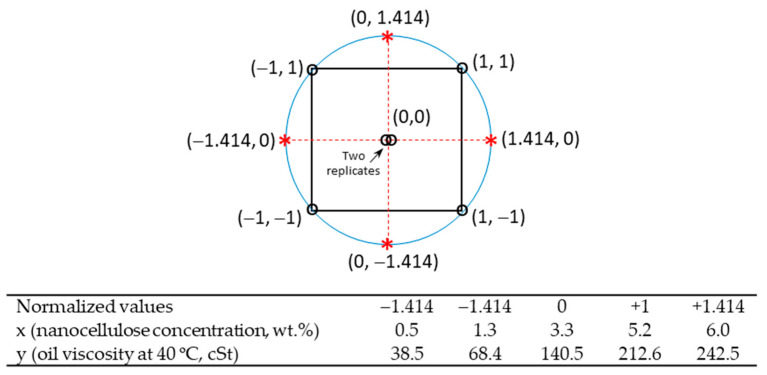
Box–Wilson Central Composite design based on a circumscribed central rotatable matrix, showing the correspondence between normalized values (levels) and actual values for the two variables (factors) of interest (nanocellulose concentration, x, and oil viscosity, y).

The research reported in this study followed a data analysis strategy based on the so-called response surface methodology (RSM) experimental design (Figure 1). Sample formulation was carried out following a Box–Wilson Central Composite design with two factors (k = 2). The method contains an imbedded factorial design with center points and a group of axial points [31]. Thus, for k = 2, the number of factorials resulted in being k^2^ = 4 (black square). The axial points (red cross), which resulted in being 2·k = 4, were at some value of α and −α on each axis. As the design was based on a circumscribed central rotatable matrix, α resulted in being k^0.5^ = 1.414 in such a way that the eight points were at the same distance from the center point. The number of central points was chosen to be 2 (two replicates of the same (0, 0) sample). Hence, a total of nine different samples were prepared. Nanocellulose concentration, ranging from 0.5 to 6 wt.%, and oil mixture kinematic viscosities at 40 °C, ranging from 38.5 cSt (pure HOSO) to 242.5 cSt (pure CO), were set as factors (i.e., study variables). As observed in Figure 1, for each factor, the upper and lower bounds are assigned normalized values of α and –α, respectively, thereby resulting in five levels (−1.414, −1, 0, 1, 1.414).

### 2.3. Preparation of Nanocellulose-Based Ecolubricants

Dispersions were prepared on a basis of 30 g vegetable oil and by following a two-step protocol as reported in [32,33]. The dispersing procedure started by mixing oil and nanocellulose with a magnetic stirrer for 45 min at 50 °C. Subsequently, sonication was applied for 40 min in a Power Sonic 405 sonication bath, for the sake of improving the quality of dispersion. A visual inspection confirmed the homogeneous dispersion of nanocelluloses in the base oils. Samples were manually stirred and then sonicated for 5 min prior to any test to ensure the characterization of fully homogeneous fluids and, thus, trustable results.

### 2.4. Rheological and Tribological Characterization of Nanocellulose-Based Ecolubricants

The viscous flow analysis of the samples was performed in a Physica MCR 301 (Anton Paar, Graz, Austria) controlled stress rheometer. Steady-state viscous flow tests were performed at 25 °C and using a smooth plate–plate geometry (50 mm diameter, 1 mm gap). The shear rate interval ranged between 0.01 and 100 s^−1^. At least two replicates were carried out for every sample.

In relation to the tribological characterization, both friction coefficient and wear scar analyses were carried out. A ball-on-three plates tribology cell coupled to a Physica MCR 501 (Anton Paar, Graz, Austria) controlled stress rheometer was used. The tribology cell was composed of a lower part with three 45° pitched steel plates (C45E-1.1191, hardness 25–30 HRC) and an upper part that holds a fixed 12.7 mm chrome steel 100Cr6 bearing ball [34]. The plates and ball were fixed on their corresponding geometries to avoid undesired vibrations and rolling, thus allowing the tests to be performed under pure sliding friction conditions. The friction coefficient was calculated as the ratio of the measured friction force to the applied normal force on the plates. At least three replicates for every operating condition and sample were carried out in order to ensure that the average values were statistically significant at the 95 % confidence level.

Friction coefficient evolution was monitored at 3 s intervals within the rotational speed range studied, from 1 to 1000 rpm (corresponding to sliding velocities in the range of 0.5–470 mm/s) at 10, 20 and 40 N (0.81– 1.29 GPa maximum Hertzian pressure) and at 25 °C. In order to conduct a more comprehensive tribological characterization, wear tests were performed at a normal load of 40 N and a fixed sliding speed of 18.8 mm/s for 1800 s and at 25 °C. Friction coefficient was registered every 9 s. Upon completion of the tests, the plates were properly cleaned with ethanol. Wear scars were then examined and recorded using an Olympus SC50 camera attached to an optical microscope, Olympus BX51 (Olympus, Tokyo, Japan).

## 3. Results and Discussion

### 3.1. Viscous Flow Behavior of Nanocellulose-Based Ecolubricants

In the shear rate interval between 1 and 100 s^−1^, all formulations studied showed Newtonian viscous flow behavior, i.e., a constant value of dynamic viscosity. Table 1 shows the average value (±standard deviation) of the samples’ dynamic viscosities, at 25 °C.

The dispersions of either CNF or CNC in CO/HOSO mixtures showed very similar viscosities. As seen in Table 1, minor differences were observed in the dynamic viscosity at 25 °C between ecolubricants containing fibrillated nanoparticles and those containing crystalline ones. In both cases, viscosity was shown to moderately increase with nanocellulose concentration for the same base oil viscosity. For example, for the same 140.5 cSt base oil (mixture with 70 wt.% CO), a viscosity increase of ca. 28% was observed when comparing the sample containing 0.5 wt.% nanocellulose (−1.414, 0) with that containing 6.0 wt.% nanocellulose (+1.414, 0). In contrast, the base oil viscosity yielded much larger changes in the lubricant viscosity for the same nanocellulose concentration. Thus, for example, for 3.3 wt.% nanocellulose, viscosity increases of more than 800% were found when comparing the sample containing neat HOSO (0, −1.414) with that containing neat CO (0, +1.414).

**Figure 2:**
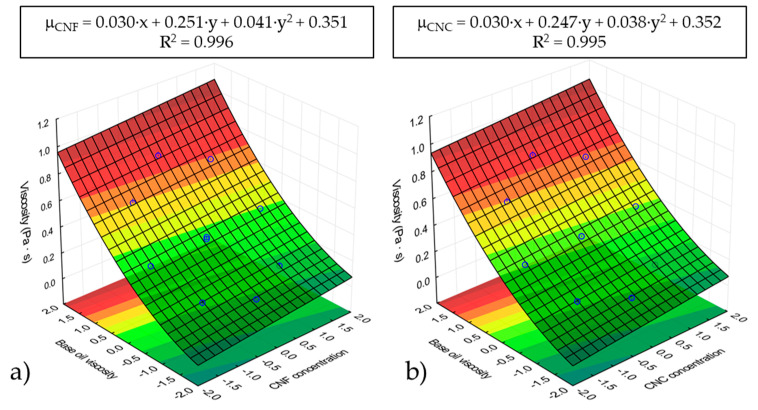
RSM analysis of viscosity values at 25 °C for (**a**) CNF-based ecolubricants and (**b**) CNC-based ecolubricants.

It is worth pointing out that the similar viscosity improvement reached with both nanocelluloses could be attributed to their similar molecular structure. Essentially, they only differ in aspect ratio, which does not seem to affect the viscous flow behavior too much. Consequently, the remarkable increase in viscosity when castor oil was used as the base oil may most probably be attributed to intermolecular H-bonding between the −OH groups present in the nanocellulose particles and those pending from the C12 at the ricinoleic fatty acid of castor oil [35,36]. Figure 2 shows the response surface of dynamic viscosity, at 25 °C, for both nanocelluloses studied as a function of normalized values of nanocellulose concentration (x) and base oil viscosity (y). Regardless of the nanocellulose type, similar fitting functions were observed. A fairly good regression of the experimental data was obtained. Dynamic viscosity was found to have a quadratic dependency on base oil viscosity (y) and a slight linear dependency on nanocellulose concentration (x).

### 3.2. Friction Behavior of Nanocellulose-Based Ecolubricants

**Figure 3:**
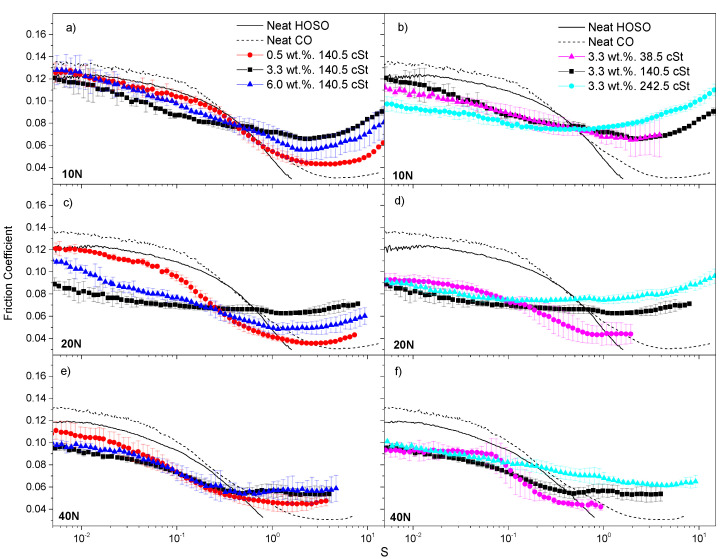
Stribeck curves for CNF-based dispersions, at 25 °C, as a function of nanocellulose concentration (**a**,**c**,**e**) and base oil viscosity (**b**,**d**,**f**) at 10, 20 and 40 N (first, second and third rows, respectively). Neat base oils are included for the sake of comparison.

**Figure 4:**
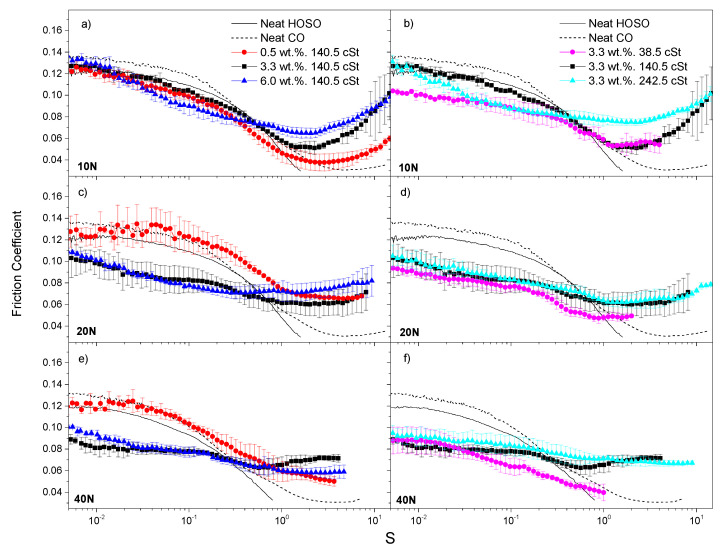
Stribeck curves for CNC-based dispersions, at 25 °C, as a function of nanocellulose concentration (**a**,**c**,**e**) and base oil viscosity (**b**,**d**,**f**) at 10, 20 and 40 N (first, second and third rows, respectively). Neat base oils are included for the sake of comparison.

An in-depth analysis of the tribological behavior is presented in this section. Figure 3 and Figure 4 depict the variation of the friction coefficient corresponding to CNF- and CNC-based ecolubricants, respectively, within the sliding velocity range studied. The data were acquired under normal forces of 10, 20 and 40 N (equivalent to mean Hertzian forces between 0.81 and 1.29 GPa) and sliding velocities ranging from 0.5 to 500 mm/s. In order to enable a direct comparison of the friction coefficient across formulations with different viscosities, abscissa values were normalized to the so-called Hersey dimensionless parameter [37]. This parameter takes into account both operation conditions (u, F_N_) and fluid viscosity (µ) in accordance with Equation (1):

(1) $$S=\frac{u\cdot \;µ}{F_{N}}$$

Figure 3 shows the Stribeck curves of CNF-based dispersions in terms of their CNF concentration dependency (Figure 3a,c,e) and oil viscosity dependency (Figure 3b,d,f). Regarding the CNF concentration dependency, Figure 3a,c,e (left column) correspond to concentration values of 0.5, 3.3 and 6.0 wt.%, respectively, and a fixed base oil viscosity of 140.5 cSt (mixture with 70 wt.% CO). Concentrations equal to or higher than 3.3 wt.% brought about, in general, a reduction in the friction coefficient along the solid friction-prevailing region (boundary regime) as compared to the neat oils, mainly at normal forces equal to or higher than 20 N. Nevertheless, the differences among the curves became smaller as the applied normal force was raised to 40 N. In all cases, the lowest CNF concentration studied, 0.5 wt.%, gave rise to the highest friction coefficient values along the boundary to mixed lubrication regions. Therefore, it is noteworthy that the addition of CNF concentrations equal to or higher than 3.3 wt.% enhanced the lubricant friction-reducing properties under solid friction conditions. On the other hand, as expected, a marked decrease in the friction coefficient was appreciated when the 0.5 wt.% CNF dispersion approached the hydrodynamic regime. As for the oil viscosity dependency, Figure 3b,d,f (right column) correspond to base oil viscosity values of 38.5, 140.5 and 242.5 cSt, respectively, and a fixed CNF concentration of 3.3 wt.%. A significant reduction in the friction coefficient within the boundary lubrication (pure solid friction) regime was noticed with the addition of 3.3 wt.% CNF as compared to the neat vegetable oils tested. This outcome reveals the friction-reducing functional properties of cellulose nanoparticles. This phenomenon was more evident at the two highest normal forces evaluated, i.e., 20 and 40 N. All along the solid friction-based lubrication region and within the base oil viscosity range studied (38.5–242.5 cSt), the friction coefficient curves remained, in general, very steady and close to each other regardless of the base oil used at a fixed CNF concentration of 3.3 wt.%. However, a noticeable decay in the friction coefficient within the mixed friction regime was observed for the ecolubricant formulated with neat HOSO (38.5 cSt), mainly at 40 N. Finally, the high viscosity of castor oil and its large affinity with nanocellulose, as explained above, yielded dispersions with much higher viscosities (Table 1), which led to an evident increase in the friction coefficient along the hydrodynamic (HD) regime, where the fluid viscosity plays a major role.

For those formulations prepared with CNC, the related Stribeck curves are shown in Figure 4. Figure 4a,c,e correspond to tests conducted under normal forces of 10, 20 and 40 N, respectively, on mixtures of 0.5, 3.3 and 6.0 wt.% CNC in a base oil with a fixed intermediate viscosity of 140.5 cSt. Again, CNC concentrations equal to or higher than 3.3 wt.% led to an outstanding friction reduction along the solid friction-prevailing regions (boundary to mixed friction regimes) at normal forces of 20 and 40 N. At 10 N, in turn, only minor differences in the friction coefficient within the CNC concentration range studied were observed as compared with both neat vegetable oils. Such a result also demonstrates the friction-reducing capacity associated with CNC under predominant solid friction conditions. Regarding the effect of base oil viscosity (Figure 4b,d,f), the behavior observed was very similar to that for CNF-based dispersions. Thus, 3.3 wt.% CNC-based ecolubricants displayed remarkable friction-reducing properties at both 20 and 40 N within both boundary and mixed lubrication regions, whatever the oil viscosity was. Under hydrodynamic lubrication conditions, the behavior was also as previously described for CNF. Hence, larger friction coefficients correlate with higher viscosities.

In general, both nanocelluloses studied showed friction-reducing capacity and, under the working conditions used in Figure 3 and Figure 4, both of them seemed to perform equally well. They might accumulate within the solid asperities, thereby efficiently reducing the actual contact area between the solid bodies [7]. Moreover, there seems to be a threshold nanocellulose concentration of around 3.3 wt.%, from which the lubricant exhibited noticeably enhanced friction-reducing performance under solid friction conditions.

**Figure 5:**
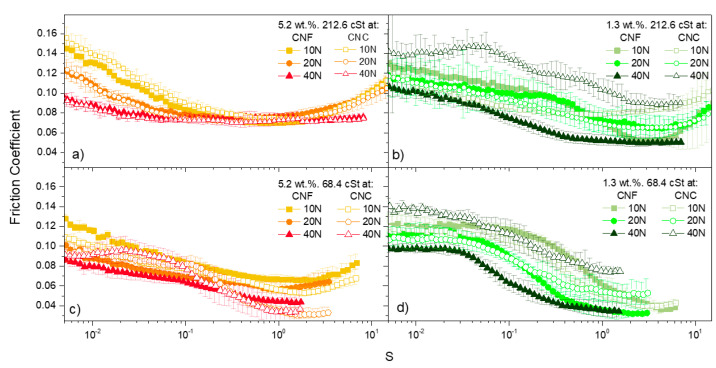
Comparison of the friction coefficient as a function of applied normal force for CNF-based (filled symbol) and CNC-based (empty symbol) ecolubricants, at 25 °C, at selected combinations of base oil viscosity and nanocellulose concentration: (**a**) 5.2 wt.% and 212.6 cSt; (**b**) 1.3 wt.% and 216.6 cSt; (**c**) 5.2 wt.% and 68.4 cSt; (**d**) 1.3 wt.% and 68.4 cSt.

Figure 5 illustrates the Stribeck curves corresponding to the two-level factorial part of the Box–Wilson Central Composite design, i.e., the black square in Figure 1, which represents the four possible combinations of the +1 and −1 levels of the two factors studied: 5.2 and 1.3 wt.% nanocellulose concentrations, respectively, and 212.6 and 68.4 cSt base oil viscosities, respectively. This enabled an easier evaluation of the combined effect of the study variables (factors). Thus, for high concentration and high kinematic viscosity (5.2 wt.% and 212.6 cSt, respectively, in Figure 5a) the friction reduction effectiveness significantly improved with increasing the applied normal force, along the solid friction-prevailing regions (boundary and mixed regions), as compared with the mixture containing 1.3 wt.% nanocellulose and the same base oil (Figure 5c). On the other hand, CNC failed (too high friction coefficient) to support a load of 40 N at a low concentration (1.3 wt.%), no matter the base oil used. Therefore, it can be pointed out that, if a low concentration lubricant is demanded, CNF seems to be a better choice than CNC, as its friction-reducing properties are further enhanced as the applied load is raised up to 40 N.

### 3.3. Wear Analysis of Nanocellulose-Based Ecolubricants

As reported above, the best friction behavior of nanocellulose particles studied was observed at high normal forces. Based on that, and in order to conduct a more comprehensive tribological characterization, an analysis of both the wear scar and the evolution with time of the friction coefficient was conducted under a normal load of 40 N and a sliding velocity of 18.8 mm/s for 1800 s and at 25 °C (room temperature). These operation conditions correspond to the mixed lubrication region (or elastohydrodynamic regime), as corroborated by previous Stribeck-like curves. Mixed lubrication is well known to be strongly influenced not only by the base oil (viscosity and composition) and additives used but also by the characteristics of the metal surface [38,39]. Therefore, the tribological properties of these nanocellulose-based ecolubricants were also examined according to the above-explained experimental design.

**Figure 6:**
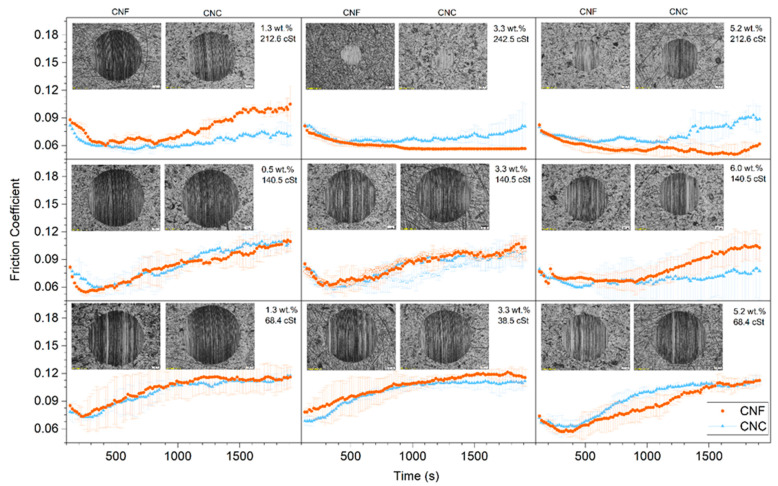
Evolution of friction coefficient with time and wear scars during wear tests at constant sliding velocity of 18.8 mm/s and normal load of 40 N for CNF-based (red curves) and CNC-based (blue curves) ecolubricants.

Figure 6 shows the evolution of the friction coefficient with time and the wear scars under mixed friction conditions. For the sake of clarity, the results are provided in the form of a 3 × 3 matrix, following the RSM described in Figure 1. As expected, the friction curves departed from the static friction coefficient value and suddenly started to decrease down to a minimum value, the so-called dynamic friction coefficient. Under the severe conditions imposed, in most cases, the friction coefficient increased with time until the test ended. Only those ecolubricants with nanocellulose concentrations equal to or higher than 3.3 wt.% and base oil viscosities equal to or higher than 212.6 cSt (mixture with 93 wt.% CO) showed a nearly constant friction coefficient all along the test. It is worth pointing out that the increase in the friction coefficient detected in the ecolubricants with the lowest oil viscosities and nanocellulose concentrations might be a sign of failure in their load-carrying capacity. In such cases, much larger wear scars were observed (Figure 6 and Table 2).

**Figure 7:**
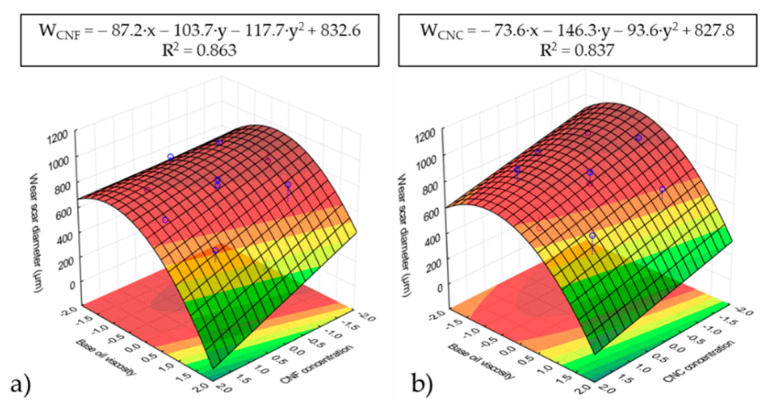
Response surface analysis (RSM) based on wear scar diameters for CNF (**a**) and CNC (**b**) ecolubricants.

**Figure 8:**
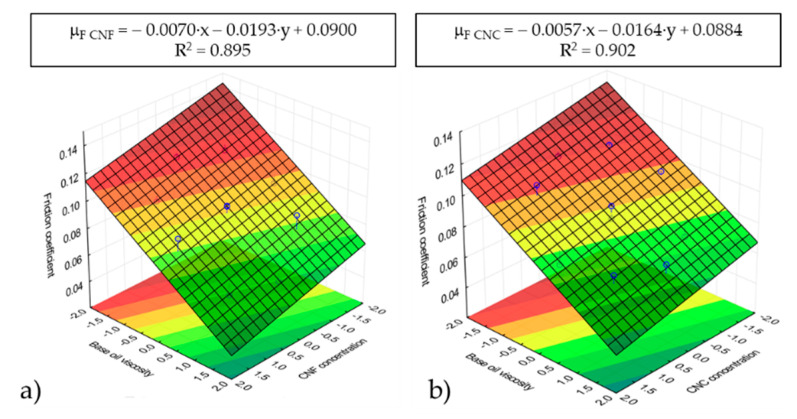
Response surface analysis (RSM) based on friction coefficients for CNF (**a**) and CNC (**b**) ecolubricants.

RSM analysis was performed on both the wear scar diameter and the average friction coefficient, and the results are shown in Figure 7 and Figure 8, respectively. In addition, the wear scars diameters are presented in Table 2, where the two neat vegetable oils used were added for the sake of comparison. As can be appreciated in Figure 7 and Figure 8, both the friction coefficient and the wear scar diameter are strongly dependent on the base oil viscosity, i.e., the base oil composition. However, a lower dependency on nanocellulose concentration was found. Thus, the higher the base oil viscosity (i.e., the higher the castor oil wt.%) was, the lower both the friction coefficient and the wear scar diameter. The friction coefficient results in Figure 8 match the base oil viscosity and the nanocellulose concentration dependencies of the dynamic viscosity corresponding to the ecolubricants studied (Figure 2). According to the theory by Hamrock and Dowson [40], higher viscosities contribute to thicker lubricant films, which significantly reduce the friction coefficient within the mixed friction regime. Hence, the existence of H-bonds between the three −OH groups present in the glucose unit of cellulose and the pending −OH at the C12 in the ricinoleic acid may have contributed to form thicker lubricant films with improved tribological performance. This outcome highlights the important contribution of the lubricant chemistry, placing special emphasis on the role of H-bonding as a compatibilizer of nanocelluloses in castor oil-rich ecolubricants. Therefore, it can be concluded that the friction coefficient reduction was enhanced by using those nanocellulose-based ecolubricants with the highest castor oil percentages.

The wear scar on the metal surface upon completion of the wear tests was also analyzed. In all cases, the presence of furrows suggests that the main wear mechanism is abrasion, in accordance with [11,41,42]. The average wear scar diameters for the different samples studied are listed in Table 2. If compared to neat castor oil (a wear scar diameter of 520 ± 13 µm), almost all ecolubricants tested showed an increase in the wear scar diameter at 40 N. Only those formulations containing 3.3 wt.% nanocellulose and a base oil viscosity of 242.5 cSt resulted in a reduction of ca. 40% and 36.7% for CNF-based (312 ± 32 µm) and CNC-based (329 ± 9 µm) ecolubricants, respectively, with respect to their base oil (neat CO). The ecolubricant with a 212.6 cSt base oil (mixture with 93 wt.% CO) resulted in a wear scar diameter of 543 ± 47 µm, very similar to that of neat CO, when the nanocellulose concentration was as high as 5.2 wt.%. Otherwise, the wear scar diameter was much higher. So, the lower the CO/HOSO ratio was, the higher the nanocellulose concentration for the lubricant to maintain its successful performance. Thus, the nanocelluloses only showed good load-carrying capacity when they were dispersed in neat castor oil or an oil mixture with high ricinoleic acid content. CO wt.% lower than 93 yielded much larger wear scar diameters. These results can be explained based on a previous study by Gao and Spikes [43], which reported the fractionation of binary mixtures, including components with different polarities in the vicinity of the metal surface. Nanocellulose-ricinoleic acid adducts formed by H-bonding are much more polar than HOSO. Hence, they might be more strongly attracted to the mating surface by van der Waals forces, contributing to wear reduction. Within the nanocellulose concentration interval studied, the addition of HOSO in the oil mixture might have constrained the effective film formation, thereby yielding a failure in the lubricant load-carrying capability.

**Figure 9:**
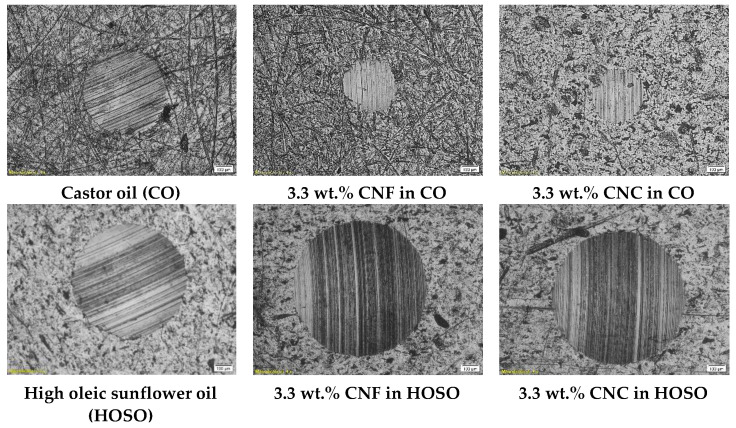
Comparative evaluation, as a function of base oil used (neat CO or neat HOSO), of wear scar diameter from wear tests performed on neat oil (**left column**), 3.3 wt.% CNF (**middle column**) and 3.3 wt.% CNC (**right column**).

Figure 9 provides concluding results on this issue. An optical microscopy visualization of wear scars on steel plates are shown for neat CO, neat HOSO and their corresponding 3.3 wt.% nanocellulose lubricants. As can be appreciated, CO-based formulations performed better in terms of load-carrying capability than HOSO-based formulations. However, further research is still required to elucidate the exact wear mechanism and tribofilm formation in these nanocellulose-based ecolubricants.

## 4. Conclusions

Both fibrillated and crystalline nanocelluloses dispersed in a highly viscous base oil, such as castor oil, were proven successful in reducing the friction coefficient. Thus, a very noticeable friction coefficient reduction within the boundary and mixed friction regions was appreciated using a formulation containing a high nanocellulose concentration (equal to or higher than 3.3 wt.%), mainly at normal forces equal to or higher than 20 N. In addition, as the applied normal force was increased, a higher nanocellulose concentration was required for the lubricant to maintain its effectiveness. Thus, ecolubricants with nanocellulose concentrations lower than 3.3 wt.% and a base oil viscosity lower than 212.6 cSt (less than 93 wt.% CO) did show evidence of failure in their load-carrying capacity at 40 N.

On the other hand, a marked decay in the friction coefficient at the onset of the fluid friction (HD) regime was appreciated when ecolubricants with the lowest nanocellulose concentrations were used. Nanocelluloses in castor oil-rich base mixtures yielded ecolubricants with too high viscosities so as to be used within the fluid friction regime. Thus, the lowest CNF and CNC concentrations studied proved to be more suitable under such working conditions. Moreover, it can be pointed out that CNF showed better friction-reducing properties than those of CNC at a low concentration and at 40 N.

Finally, the ecolubricants’ tribological behavior (evaluated by both the friction coefficient time evolution and the wear scar diameter under mixed lubrication conditions) was highly dependent on the base oil, while a lower dependency on nanocellulose concentration was found. Thus, the higher the CO wt.% in the oil mixture was, the lower both the friction coefficient and the wear scar diameter. The existence of H-bonds between the cellulose nanoparticles and the ricinoleic acid contributed to highly viscous formulations, which promoted friction coefficient reduction within the mixed friction regime. Moreover, wear scar reduction was explained on the basis of fractionation of the lubricant in the vicinity of the mating surface and van der Waals attraction of the most polar components, ricinoleic and nanocellulose. Wear scar diameter reductions of around 40% and 36.7% were achieved with 3.3 wt.% CNF and CNC, respectively, in castor oil. However, a weakened load-carrying capability was found with increasing the HOSO wt.% in the oil mixture.

Even so, more tests will be needed in the future with a view to exploring the exact wear mechanism and tribofilm formation in these nanocellulose-based ecolubricants.

## Figures and Tables

**Table 1:** Average dynamic viscosity values, at 25 °C, for the CNF- and CNC-based dispersions.

Sample Code	Nanocellulose Concentration (wt.%)	Base Oil Viscosity (cSt) [ ] *	CNF	CNC
			µ (Pa·s)	µ (Pa·s)
0, 0A	3.3	140.5 [70]	0.338 ± 0.006	0.347 ± 0.007
0, 0B	3.3	140.5 [70]	0.353 ± 0.005	0.346 ± 0.005
+1.414, 0	6.0	140.5 [70]	0.398 ± 0.007	0.400 ± 0.014
−1.414, 0	0.5	140.5 [70]	0.312 ± 0.002	0.313 ± 0.004
0, +1.414	3.3	242.5 [100]	0.783 ± 0.020	0.767 ± 0.010
0, −1.414	3.3	38.5 [0]	0.082 ± 0.006	0.085 ± 0.009
+1, +1	5.2	212.6 [93]	0.696 ± 0.012	0.696 ± 0.012
+1, −1	5.2	68.4 [31]	0.148 ± 0.002	0.145 ± 0.001
−1, +1	1.3	212.6 [93]	0.597 ± 0.014	0.589 ± 0.006
−1, −1	1.3	68.4 [31]	0.129 ± 0.005	0.132 ± 0.007

* CO wt.% in the vegetable oil CO/HOSO mixtures.

**Table 2:** Wear scar diameters obtained from wear tests at constant sliding velocity of 18.8 mm/s and normal load of 40 N for both nanocellulose dispersions studied.

Sample	Nanoparticle Concentration (wt.%)	Base Oil Viscosity (cSt)	CNF Diameter (μm)	CNC Diameter (μm)
Castor oil	0	242.5	520 ± 13 *	520 ± 13 *
HOSO	0	38.5	689 ± 29 *	689 ± 29 *
0, 0A	3.3	140.5	843 ± 23	858 ± 54
0, 0B	3.3	140.5	812 ± 34	773 ± 51
+1.414, 0	6.0	140.5	711 ± 24	619 ± 81
−1.414, 0	0.5	140.5	925 ± 14	948 ± 65
0, +1.414	3.3	242.5	312 ± 32	329 ± 9
0, −1.414	3.3	38.5	843 ± 13	821 ± 31
+1, +1	5.2	212.6	543 ± 47	634 ± 45
−1, +1	1.3	212.6	806 ± 10	722 ± 44
+1, −1	5.2	68.4	727 ± 30	886 ± 38
−1, −1	1.3	68.4	860 ± 29	922 ± 35

* Neat vegetable oil without CNF or CNC nanoparticles.

## Data Availability

The data presented in this study are available on request from the corresponding author.
